# Protein arginine methyltransferase 5 is a key regulator of the MYCN oncoprotein in neuroblastoma cells

**DOI:** 10.1016/j.molonc.2014.10.015

**Published:** 2014-11-15

**Authors:** Ji Hyun Park, Marianna Szemes, Gabriella Cunha Vieira, Zsombor Melegh, Sally Malik, Kate J. Heesom, Laura Von Wallwitz-Freitas, Alexander Greenhough, Keith W. Brown, Y. George Zheng, Daniel Catchpoole, Michael J. Deery, Karim Malik

**Affiliations:** ^1^ Cancer Epigenetics Laboratory University of Bristol, Bristol BS8 1TD, UK; ^2^ Department of Cellular Pathology, Southmead Hospital, Bristol, UK; ^3^ Department of Biochemistry, University of Bristol, Bristol BS8 1TD, UK; ^4^ Colorectal Cancer Laboratory, School of Cellular & Molecular Medicine University of Bristol, Bristol BS8 1TD, UK; ^5^ Department of Chemistry, Georgia State University, Atlanta, GA 30302-4098, USA; ^6^ The Kids Research Institute, The Children's Hospital at Westmead, Westmead, New South Wales 2145, Australia; ^7^ Cambridge Centre for Proteomics, Cambridge Systems Biology Centre, Department of Biochemistry, University of Cambridge, Cambridge, UK

**Keywords:** Neuroblastoma, MYCN, PRMT5, Arginine methylation

## Abstract

Approximately half of poor prognosis neuroblastomas (NBs) are characterized by pathognomonic MYCN gene amplification and MYCN over‐expression. Here we present data showing that short‐interfering RNA mediated depletion of the protein arginine methyltransferase 5 (PRMT5) in cell‐lines representative of NBs with MYCN gene amplification leads to greatly impaired growth and apoptosis. Growth suppression is not apparent in the MYCN‐negative SH‐SY5Y NB cell‐line, or in two immortalized human fibroblast cell‐lines. Immunoblotting of NB cell‐lines shows that high PRMT5 expression is strongly associated with MYCN‐amplification (P < 0.004, Mann–Whitney U‐test) and immunohistochemical analysis of primary NBs reveals that whilst PRMT5 protein is ubiquitously expressed in the cytoplasm of most cells, MYCN‐amplified tumours exhibit pronounced nuclear PRMT5 staining. PRMT5 knockdown in MYCN‐overexpressing cells, including the SHEP‐21N cell‐line with inducible MYCN expression leads to a dramatic decrease in MYCN protein and MYCN‐associated cell‐death in SHEP‐21N cells. Quantitative gene expression analysis and cycloheximide chase experiments suggest that PRMT5 regulates MYCN at a post‐transcriptional level. Reciprocal co‐immunoprecipitation experiments demonstrated that endogenous PRMT5 and MYCN interact in both SK‐N‐BE(2)C and NGP cell lines. By using liquid chromatography – tandem mass spectrometry (LC‐MS/MS) analysis of immunoprecipitated MYCN protein, we identified several potential sites of arginine dimethylation on the MYCN protein. Together our studies implicate PRMT5 in a novel mode of MYCN post‐translational regulation and suggest PRMT5 plays a major role in NB tumorigenesis. Small‐molecule inhibitors of PRMT5 may therefore represent a novel therapeutic strategy for neuroblastoma and other cancers driven by the MYCN oncogene.

## 1 Introduction

Neuroblastoma (NB) is an embryonal malignancy arising from the sympathetic nervous system and is one of the commonest solid tumours of childhood. The development of NB is believed to be contingent on defective differentiation of neural crest cells, although the precise cell of origin remains unknown. Several genetic markers are found in poor prognosis NB, including loss of chromosome 1p, loss of 11q, gain of 17q and, most notably, amplification of the *MYCN* proto‐oncogene which occurs in about 50% of high‐risk NBs (∼20% of total NBs) (Brodeur, 2003; Maris et al., 2007). Importantly, in some tumours, MYCN protein levels can also be very high without gene amplification or high mRNA levels, implicating other factors that might stabilize MYCN at the protein level; as expected, high MYCN protein is closely associated with very poor prognosis (Valentijn et al., 2012). MYCN is a classical oncogenic transcription factor and its aberrant expression is thought to deregulate neuroblast differentiation programmes by a variety of means, including recruitment of gene repressing components of the epigenetic machinery, such as histone deacetylases (Iraci et al., 2011) and Polycomb proteins such as EZH2 (Corvetta et al., 2013), the latter being a histone methyltransferase (HMT) independently shown to repress tumour suppressor genes in neuroblastoma (Wang et al., 2012). Genes repressed by MYCN include neurotrophic tyrosine kinase receptor (*NTKR*) and nerve growth factor receptor (*NGFR*) (Iraci et al., 2011), and alleviating these blocks is conducive to triggering apoptosis.

The possibilities of neuroblastoma therapeutics based on targeting of MYCN have advanced recently, notably with the demonstration that small molecule inhibitors targeting Aurora kinase A such as Alisertib (MLN8237) can trigger depletion of MYCN protein in NB cell‐lines and in the murine model of NB, leading to NB growth inhibition and tumour regression. MYCN destabilisation is not contingent on Aurora kinase A enzymatic activity, but rather on its conformation and ability to protect MYCN from FBXW7‐mediated degradation (Otto et al., 2009; Brockmann et al., 2013). Another promising class of inhibitor targeting the MYC family is exemplified by JQ‐1, which acts by inhibiting bromodomain and extra terminal domain (BET) family of proteins and thereby curtailing *MYCN* transcription (Puissant et al., 2013).

Given the increasing evidence for the importance of gene repression by histone modifications in neuroblastoma tumorigenesis cited above, we assessed another histone methyltransferase (HMT) known to catalyse repressive epigenetic modifications in a variety of cancers, specifically protein arginine methyltransferase 5 (PRMT5). PRMT5 is active in chromatin remodelling complexes, and catalyses symmetric dimethylation of the histone 3 tail at arginine 8 (H3R8) and histone 4 arginine 3 (H4R3), and is one of two “type II” arginine methyltransferases, the other being PRMT7 (Karkhanis et al., 2011; Yang and Bedford, 2013). PRMT5 over‐expression in a variety of cancers is thought to silence tumour suppressor genes (TSGs) such as *ST7* (suppression of tumorigenicity 7) (Pal et al., 2004) and genes encoding RB family proteins (Chung et al., 2013; Yang and Bedford, 2013). PRMT5 also facilitates gene silencing with DNA methyltransferase 3A (Zhao et al., 2009) and the Polycomb repressor complex (Chung et al., 2013). As well as TSG silencing, PRMT5‐mediated methylation of non‐histone proteins is a further alternative oncogenic modality for PRMT5. This is exemplified by Programmed cell death 4 (PDCD4) protein in breast cancer, which shows increased tumorigenicity when over‐expressed with PRMT5 in an orthotopic model of breast cancer (Powers et al., 2011). Other proteins post‐translationally methylated by PRMT5 include the transcription factors p53 (Jansson et al., 2008), NF‐κB (Wei et al., 2013) and E2F‐1 (Cho et al., 2012; Zheng et al., 2013).

In this report we provide evidence showing that PRMT5 is involved in poor prognosis NB, and is a key post‐translational regulator of the MYCN oncoprotein. PRMT5 inhibition may therefore represent a novel alternative for treatment of NB and other cancers driven by the MYCN oncoprotein.

## 2 Materials & methods

### 2.1 Cell Culture, transfections and protein/RNA preparation

Neuroblastoma cell lines used in this study were purchased from Deutsche Sammlung von Mikroorganismen und Zellkulturen (DSMZ) or kindly provided by The Children's Oncology group (http://www.cogcell.org), Manfred Schwab (German Cancer Research Center), Robert Ross, (Fordham University), Carmel McConville, (University of Birmingham), Pramila Ramani (University of Bristol). Cells were cultured in Dulbecco's modified eagle's medium (DMEM):F12‐HAM (Sigma) supplemented with 10% foetal bovine serum (PAA Cell Culture), 200 mM l‐Glutamine, 100 mM penicillin, 0.1 mg/mL streptomycin (Sigma) and 1% (v/v) non‐essential amino acids (Life technologies). SHEP‐Tet21N cells were cultured in RPMI 1640 supplemented with 10% tetracycline‐free foetal bovine serum (Life technologies) with tetracycline added to 1 μg/ml. Human fibroblast lines were made from primary human fibroblasts from the Coriell Institute Cell Repository stably transfected with human telomerase reverse transcriptase (kindly supplied by Grant Stewart, University of Birmingham), and were grown in DMEM with 10% foetal bovine serum. All cells were mycoplasma free, and cultured as adherent monolayers at 37 °C with 5% CO_2_.

For short‐interfering RNA (siRNA) treatments, exponentially growing cells were reverse‐transfected using Lipofectamine 2000 (Life technologies) at a final concentration of 25–50 nM according to the manufacturer's instructions. After 48–72 h, cells were lysed for protein in Cell signalling lysis buffer (New England Biolabs), or for RNA in QIAzol (QIAGEN). Total RNA was DNAsed and purified using the miRNeasy Mini Kit (QIAGEN). Details of siRNAs (Sigma) are given in Supplemental Table 1.

Cell counts were done in quadruplicate and repeated at least twice using a Countess cell‐counter (Invitrogen). Photographs were taken with a Leica microscope camera using Spot Advanced software.

### 2.2 Cycloheximide (CHX) chase experiments and inhibitor studies

To assess protein stability, cells were treated with 50 μg/ml cycloheximide (Sigma) following siRNA transfection for 72 h. At the given timepoints, cells were washed in ice‐cold PBS and prepared for SDS‐polyacrylamide gel electrophoresis (SDS‐PAGE) as below. For inhibition of apoptosis, siRNA transfected cells were treated with 10 μM caspase inhibitor Q‐VD‐OPh (QVD, Sigma) 24 h after knockdown.

### 2.3 Co‐immunoprecipitations

Cells were lysed in Cell Signalling Lysis buffer complemented with Complete Mini Protein inhibitors (Roche), 5 mM dithiothreitol and 10% (v/v) glycerol, and sonicated using a Bioruptor (Diagenode). The co‐immunoprecipitations were carried out according to the manufacturer's protocol (Dynabeads^®^ Protein G Immunoprecipitation Kit, Life Sciences Technology). Briefly, for each immunoprecipitation 8 μg antibody was conjugated to 50 µL Protein‐G magnetic beads (Millipore). After preclearing with normal mouse or rabbit IgG, 500 μg protein lysate was incubated overnight with the antibody‐coated beads at 4 °C with rotation. Beads were washed twice in IP lysis buffer and once in PBS and boiled in 1× Laemmli sample buffer (312.5 mM Tris–HCl pH 7.4, 50% (v/v) glycerol, 10% (w/v) SDS (Sodium Dodecyl Sulphate; Fisher Scientific), 5% (v/v) 2‐Mercaptoethanol (Sigma) before SDS‐PAGE.

### 2.4 Immunoblotting and immunohistochemistry

Immunoblotting and immunohistochemistry were performed as described previously (Szemes et al., 2013). Neuroblastoma sections were from archival tissues collected at The Children's Hospital at Westmead Histopathology Department since 1950, and assembled into a tissue microarray containing 49 cases. Full details of the TMA are published (Chetcuti et al., 2014). For immunohistochemistry primary anti‐PRMT5 antibody was used at a dilution of 1:300. Details of other antibodies and dilutions used are given in Supplemental Table 1.

### 2.5 Gene expression analysis

Quantitative real‐time reverse transcriptase‐PCR (qRT‐PCR) was performed as previously described (Szemes et al., 2013). Details of primers used (Sigma) are given in Supplemental Table 1.

### 2.6 Flow cytometry analysis

For analysis of DNA content, cells were harvested, rinsed in phosphate‐buffered saline (PBS), permeabilised in ice cold 70% ethanol, and stored at 4 °C overnight. Before fluorescence‐activated cell‐sorting (FACs), cells were washed with PBS, treated with 100 μg/ml RNase A (Qiagen) and then stained with 50 μg/ml propidium iodide (Sigma) for 30 min in the dark at 37 °C. DNA content of propidium iodide stained cells was measured on a LSRFortessa X‐20 (BD Biosciences) and data analysed using ModFit LT (Verity Software House). All assays were conducted on triplicate samples unless otherwise stated.

### 2.7 Liquid chromatography – tandem mass spectrometry (LC‐MS/MS) analysis

MYCN was immunoprecipitated as described above from control and PRMT5 siRNA‐treated SK‐N‐BE(2)C cells. Prior to harvest, cells were treated with 20 μg/ml MG132 for 2 h to circumvent possible proteosomal degradation of unmethylated MYCN. Protein was fractionated by SDS‐PAGE using SYPRO^®^ Ruby (Invitrogen) staining and visualised using a Typhoon 9400 variable mode imager (GE Healthcare). Proteins equal or above the MYCN band were excised and subjected to in‐gel tryptic digestion using a Pro‐Gest automated digestion unit (Digilab UK). The resulting peptides were fractionated using a Dionex UltiMate 3000 nano HPLC system in line with an LTQ‐Orbitrap Velos mass spectrometer (Thermo Scientific). In brief, peptides in 1% (v/v) formic acid were injected onto an Acclaim PepMap C18 nano‐trap column (Dionex). After washing with 0.5% (v/v) acetonitrile 0.1% (v/v) formic acid peptides were resolved on a 250 mm × 75 μm Acclaim PepMap C18 reverse phase analytical column (Dionex) over a 150 min organic gradient, using 7 gradient segments (1–6% solvent B over 1 min, 6–15% B over 58 min, 15–32%B over 58 min, 32–40%B over 3 min, 40–90%B over 1 min, held at 90%B for 6 min and then reduced to 1%B over 1 min) with a flow rate of 300 nl min^−1^. Solvent A was 0.1% formic acid and Solvent B was aqueous 80% acetonitrile in 0.1% formic acid. Peptides were ionized by nano‐electrospray ionization at 2.1 kV using a stainless steel emitter with an internal diameter of 30 μm (Thermo Scientific) and a capillary temperature of 250 °C. Tandem mass spectra were acquired using an LTQ‐Orbitrap Velos mass spectrometer controlled by Xcalibur 2.1 software (Thermo Scientific) and operated in data‐dependent acquisition mode. The Orbitrap was set to analyse the survey scans at 60,000 resolution (at *m/z* 400) in the mass range *m/z* 300–2000 and the top twenty multiply charged ions in each duty cycle selected for MS/MS in the LTQ linear ion trap. Charge state filtering, where unassigned precursor ions were not selected for fragmentation, and dynamic exclusion (repeat count, 1; repeat duration, 30 s; exclusion list size, 500) were used. Fragmentation conditions in the LTQ were as follows: normalized collision energy, 40%; activation q, 0.25; activation time 10 ms; and minimum ion selection intensity, 500 counts.

The raw data files were processed and converted into mgf text files using Proteome Discoverer software v1.2 (Thermo Scientific). The mgf files were submitted to the Mascot search algorithm (Matrix Science, London UK) and searched against the UniProt human database (May 2014 9606_2014_05, 156503 sequences; 55255217 residues), using the following settings: peptide precursor mass tolerance was set at 10 ppm, and MS/MS tolerance was set at 0.8 Da. Search criteria included carbamidomethylation of cysteine (+57.0214) as a fixed modification and oxidation of methionine (+15.9949) and methylation of arginine (+14.0157, +28.0314) and lysines (+14.0157, +28.0314, +42.0471) as variable modifications. Searches were performed using trypsin as the enzyme with one missed cleavage. The reverse database search option was enabled and all peptide data was filtered to satisfy false discovery rate (FDR) of 5%. Any modified peptides which were identified by Mascot were manually verified by examining the raw MS/MS data.

### 2.8 Quantitative and statistical analyses

Gel images were quantified using Image J software, and Student's *t*‐test and Mann–Whitney tests were used for statistical analysis.

## 3 Results and discussion

### 3.1 PRMT5 knockdown leads to apoptosis of neuroblastoma cells

**Figure 1:**
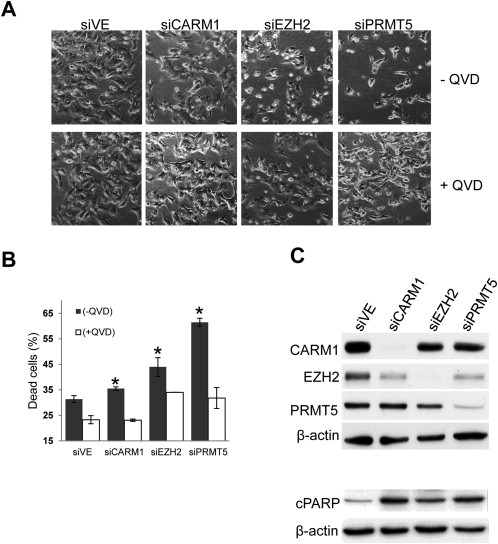
PRMT5 knockdowns induce apoptosis in SK‐N‐BE(2)C cells. (A) Short‐interfering RNAs (siRNAs) targeting CARM1/PRMT4, EZH2 and PRMT5 induced varying degrees of SK‐N‐BE(2)C growth inhibition and cell death after 72 h incubation. The negative control siRNA is also shown (siVE). Cell death was rescuable using QVD. (B) Percentage of dead cells per siRNA treatment are shown without (black bars) and with (white bars) QVD treatment. Significant differences are shown by asterisks (P < 0.05). (C) Verification of knockdowns (upper panel) and apoptosis by immunoblotting for cleaved PARP (cPARP) (lower panel).

**Figure 2:**
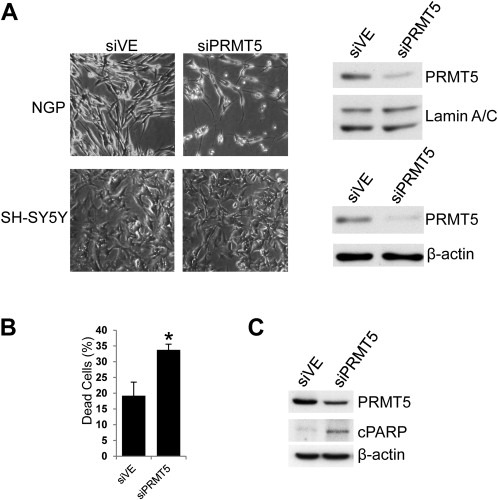
PRMT5 knockdown induces apoptosis in the NGP cell‐line, but not in the SH‐SY5Y cell‐line. (A) Cell death triggered by PRMT5 in NGP cells, together with knockdown verification by immunoblotting. No phenotypic changes were observed in SH‐SY5Y cells. (B) Quantification of NGP cell death by cell counting, the asterisk signifying significance (P < 0.05). (C) Confirmation of increased cleaved PARP (cPARP) by immunoblotting following PRMT5 knockdown.

We evaluated the effect of short‐interfering RNA (siRNA) – mediated PRMT5 depletion on the SK‐N‐BE(2)C cell‐line, representative of the poor prognosis subset of NB, together with two other HMTs known to be involved in cancer. Knockdown of CARM1 (PRMT4), EZH2 and PRMT5 all induced some cell death in SK‐N‐BE(2)C cells, but PRMT5 depletion triggered a significantly greater effect with approximately double the cell death compared to the negative control siRNA, and substantially greater death than either CARM1 or EZH2 knockdowns. Treatment of knockdowns with the caspase inhibitor Q‐VD‐OPh rescued cell death, and apoptosis was further confirmed by immunoblotting for poly(ADP‐ribose) polymerase‐1 (PARP‐1) cleavage (Figure 1A–C). PRMT5 depletion also induced cell death in NGP cells, but not in SH‐SY5Y NB cells or normal fibroblasts, despite efficient knockdowns (Figure 2 and Figure S1). PRMT5 depletion also had little effect on SHEP cells without high MYCN (see below). Increased cell death and PARP cleavage consistent with apoptosis was also apparent in NGP cells transfected with PRMT5 siRNA (Figure 2B and C).

### 3.2 PRMT5 expression correlates with MYCN in primary neuroblastomas

**Figure 3:**
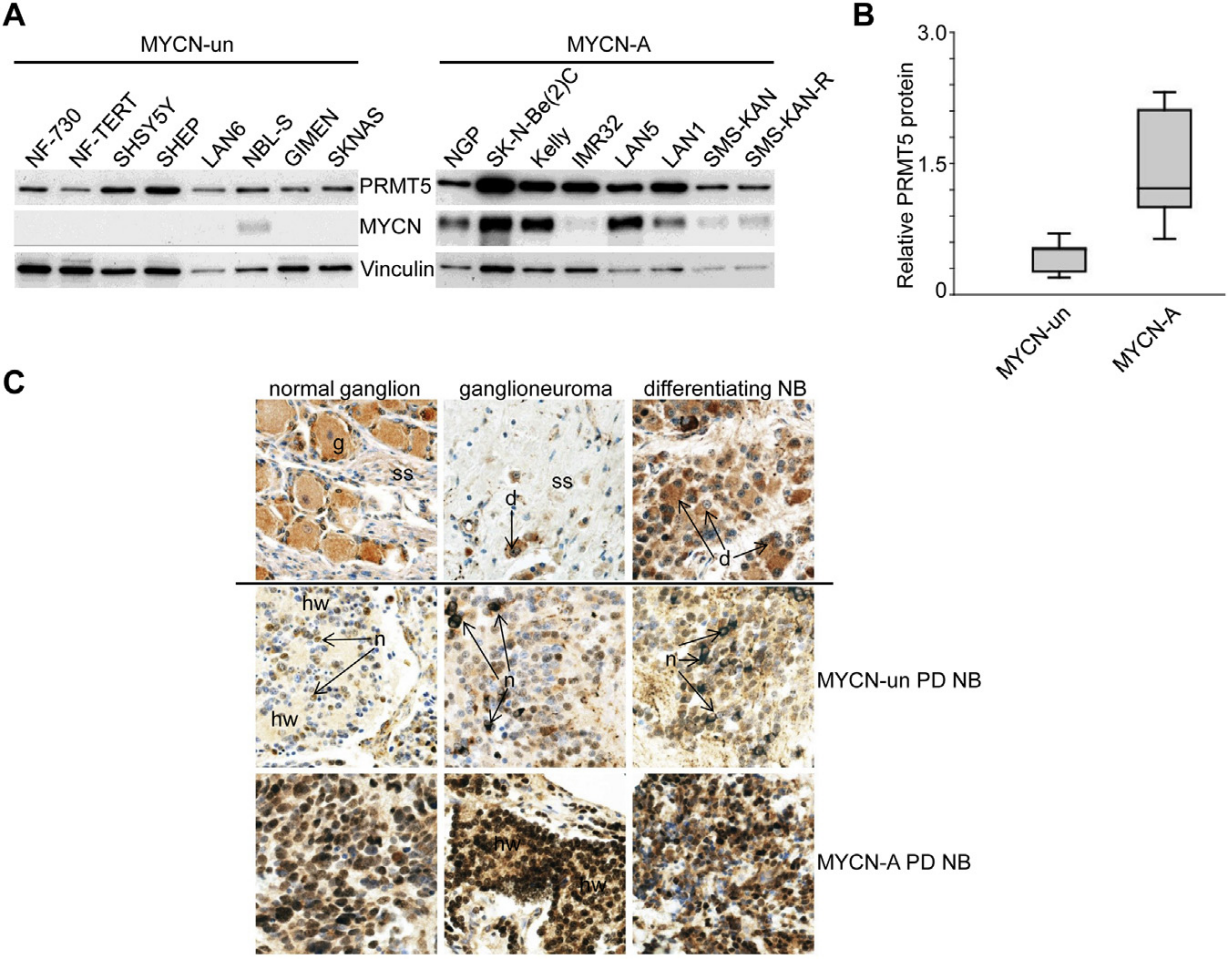
PRMT5 and MYCN protein expression correlations in neuroblastoma. (A) Immunoblotting of PRMT5 in NB cell‐lines without MYCN amplification (MYCN‐un) and cells with amplification (MYCN‐A). Vinculin is used as a loading control. (B) A box plot showing PRMT5 levels normalised to vinculin in cell‐lines demonstrates a significant over‐expression of PRMT5 in MYCN‐A lines (P < 0.004, Mann–Whitney U test). (C) Immunohistochemical staining of NB sections for PRMT5 protein: the top row shows a normal ganglion (g) with cytoplasmic PRMT5 staining arrowed, and Schwannian stroma (ss), followed by a ganglioneuroma with differentiating neuroblasts (d) with cytoplasmic PRMT5 staining arrowed, and finally a differentiating NB, again with cytoplasmic PRMT5. The second row shows differentiating neuroblastomas without MYCN amplification showing predominantly cytoplasmic PRMT5 expression. Neuroblasts (n) and Homer Wright rosettes (hw) are indicated. The third row shows poorly differentiated neuroblastomas with MYCN amplification displaying intense nuclear PRMT5 staining. For PRMT5 immunohistochemistry controls, we used skeletal muscle which is negative for PRMT5 and normal prostate where PRMT5 expression has been reported to be strong in the nucleus of the epithelial cells (Gu et al., 2012) (Figure S3).

Both SK‐N‐BE(2)C and NGP cells are known to have *MYCN* amplification and high MYCN protein expression, whereas SH‐SY5Y cells do not, suggesting a relation between PRMT5 and MYCN expression. Examination of PRMT5 expression in NB cell‐lines confirmed that MYCN over‐expression was associated with higher PRMT5 protein levels (*P* = 0.004, Mann–Whitney *U* test) (Figure 3A–B). At the RNA level, *in silico* analysis shows that high *PRMT5* transcripts also correlate with *MYCN* amplification status (*P* = 2.0e^−08^) and with poor overall prognosis (*P* = 2.2e^−08^) (Figure S2A). Notably, however, elevated *PRMT5* mRNA levels were also associated with poor outcome in *MYCN*‐unamplified tumours (*P* = 1.7e^−06^) (Figure S2B), alluding to a possible MYCN‐independent oncogenic role for PRMT5 in NB. For example, PRMT5 has been shown to regulate cell‐cycle progression (Scoumanne et al., 2009) and modify other proteins involved in NB tumorigenesis, such as E2F‐1 and p53 (Jansson et al., 2008; Cho et al., 2012).

We further examined PRMT5 protein expression in primary NB tumour sections by immunohistochemistry. As shown in Figure 3C, diffuse cytoplasmic staining was observed with the PRMT5 antibody in normal ganglion in both ganglion and satellite cells. Similarly, in a ganglioneuroma, tumour cells showed cytoplasmic PRMT5 with no nuclear staining. This pattern was also observed in a differentiating neuroblastoma without *MYCN* amplification. In poorly differentiated neuroblastomas lacking *MYCN* amplification, 57% (*n* = 14) showed predominant nuclear staining whereas 43% showed equal or more staining in the cytoplasm. In striking contrast, poorly differentiated MYCN‐amplified tumours showed intense nuclear PRMT5 staining together with weaker cytoplasmic staining. In total, 89% (*n* = 9) poorly differentiated MYCN‐amplified neuroblastomas showed predominant nuclear staining, whereas only 22% of a group including ganglioneuromas, ganglioneuroblastomas and differentiating neuroblastoma (*n* = 9) showed equivalent or increased nuclear staining compared to cytoplasmic staining. Together these expression analyses strongly suggest a PRMT5‐MYCN oncogenic axis in NB.

The nuclear co‐expression of PRMT5 and MYCN in poor prognosis NBs alludes to a co‐operative function for these proteins in gene regulation, including the possible targeting of PRMT5 to gene promoters by MYCN. We therefore tested whether *ST7* and *RBL2* genes might also be targeted by PRMT5/MYCN in NB. These genes are established PRMT5‐silenced TSGs in other cancers (Pal et al., 2004; Chung et al., 2013), However, little or no change was observed in their transcript levels with either PRMT5 or MYCN knockdowns. We also assessed some genes encoding regulators of neuroblast differentiation and cell death which are known to be repressed by MYCN together with histone deacetylase 1, specifically *NTRK* and *NGFR* (Iraci et al., 2011). Whilst both genes were reactivated following MYCN knockdown, only *NGFR* mRNA was markedly increased (over 3‐fold) with PRMT5 knockdown in NGP cells, with little effect being apparent in SK‐N‐BE(2)C cells (Figure S4A). Thus PRMT5 and MYCN may co‐operate in silencing of *NGFR* in NGP cells. A comprehensive analysis of PRMT5‐and MYCN‐regulated gene signatures and promoter recruitment will be required to fully assess co‐operative gene regulation exerted by PRMT5 and MYCN.

### 3.3 PRMT5 is a key regulator of the MYCN protein

**Figure 4:**
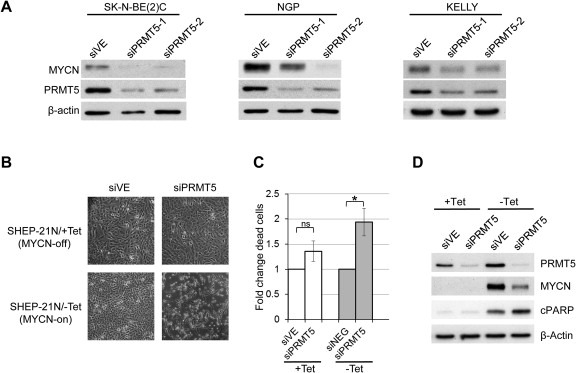
MYCN depletion triggered by PRMT5 knockdown. (A) Immunoblot analysis showing MYCN depletion in SK‐N‐BE(2)C, NGP and Kelly NB cell‐lines following transfection with two independent PRMT5 siRNAs, negating the possibility of off‐target effects. (B) PRMT5 knockdown in the SHEP‐Tet21N cell‐line harbouring inducible MYCN expression. MYCN is switched off in the presence of tetracycline (+Tet) and induced after its removal (−Tet). Cell death is clearly visible in the MYCN‐on cells after PRMT5 knockdown. (C) Cell counts showing significantly increased cell death (asterisked, P < 0.005, Student's t‐test) after PRMT5 knockdown in cells induced to express MYCN. A statistically insignificant change (not significant, ns) is observed in MYCN‐off cells. (D) Immunoblots of SHEP‐Tet21N cells demonstrating that PRMT5 also affects MYCN expressed from the inducible transgene. Depletion is accompanied by an increase in cleaved PARP indicative of apoptosis.

To further investigate the strong association between PRMT5 and MYCN status, we next examined the effect of PRMT5 knockdown on MYCN in SK‐N‐BE(2)C, NGP and Kelly cell‐lines, all of which have *MYCN* amplification. As shown in Figure 4A, PRMT5 depletion was accompanied by a dramatic decrease of MYCN protein with two independent PRMT5 siRNAs, thereby negating the possibility of off‐target effects. Notably, the PRMT5‐dependent MYCN decrease in NGP cells suggests an alternative mechanism for PRMT5 compared to the BET domain inhibitor JQ‐1, which did not affect MYCN in NGP cells (Puissant et al., 2013). *MYCN* transcript levels were only moderately decreased accompanying PRMT5 knockdown (Figure S4B), suggesting that PRMT5 mediates MYCN regulation at post‐transcriptional levels, although we cannot currently exclude composite effects including indirect transcriptional regulation, for example by PRMT5 depletion leading to reactivation of epigenetically silenced MYCN repressor proteins or microRNAs.

We also evaluated PRMT5 knockdown in the SHEP‐Tet21N cell‐line, which has inducible MYCN expression following removal of tetracycline (Figure 4B–D). PRMT5 depletion led to reduced MYCN and a statistically significant increase in cell‐death in MYCN‐overexpressing cells (*P* < 0.0.005) whereas cells without induced MYCN did not show a significant increase in cell death. Immunoblotting also showed an increase in cleaved PARP indicative of apoptosis accompanying PRMT5 knockdown in the induced cells.

**Figure 5:**
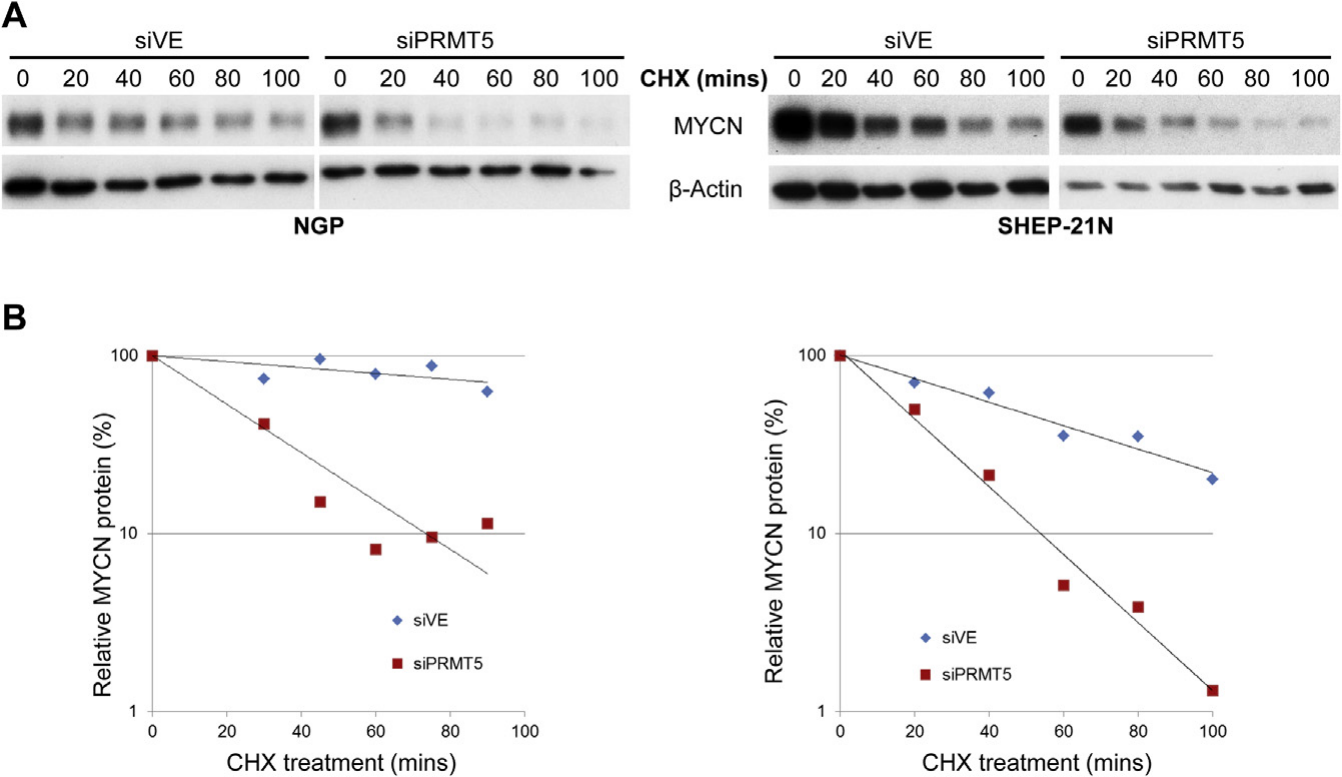
PRMT5 regulates MYCN stability. (A) Cycloheximide (CHX) treated samples analysis following negative control (siVE) or PRMT5 (siPRMT5) knockdown in NGP cells (left) and SHEP‐21N cells (right). (B) Plots of densitometric quantification of MYCN protein stability assays. MYCN levels are normalised relative to β‐actin.

In order to assess PRMT5 influence on post‐translational MYCN stability, we conducted cycloheximide chase assays on PRMT5 siRNA transfected cells. As shown in Figure 5A‐B, MYCN is turned over with a half‐life of approximately 90 min in NGP cells transfected with negative control siRNA, but the half‐life is dramatically decreased to approximately 22 min in cells transfected with PRMT5 siRNA. Similarly, in induced SHEP‐Tet21N cells, MYCN protein exhibits a half‐life of approximately 46 min, which is reduced to approximately 18 min after PRMT5 knockdown.

**Figure 6:**
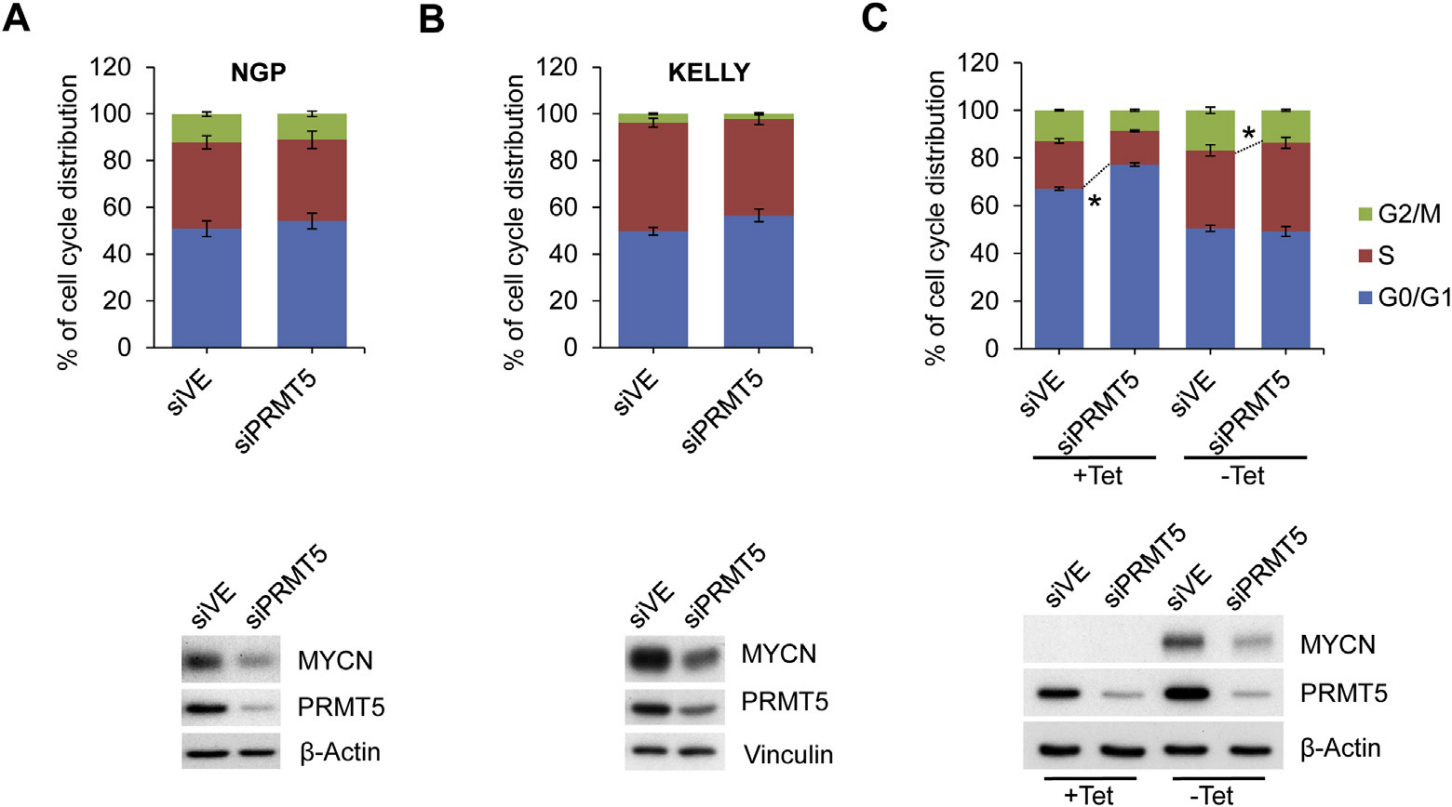
Cell‐cycle changes accompanying PRMT5 knockdowns. Following PRMT5 knockdown, moderate increases in G1 were apparent in NGP and Kelly cell‐lines, and statistically significant G1‐arrest was apparent in uninduced SHEP‐Tet21N cells (asterisked, P < 0.0001, Student's t‐test). In induced SHEP‐Tet21N cells, no G1‐arrest was evident; however a significant G2/M‐phase decrease was apparent (asterisked, P < 0.005). The latter analysis was done with duplicate samples.

In neuronal progenitor cells, MYCN is proteosomally regulated during mitosis (Sjostrom et al., 2005), so we investigated whether the PRMT5‐depletion effect on MYCN might be indirect and attributable to solely to G1 cell‐cycle arrest. Whilst a degree of G1 arrest was apparent in NGP and Kelly cells after PRMT5 knockdown, it was below statistical significance (Figure 6A and B). Furthermore, G1 arrest was not observed after PRMT5 knockdown in SHEP‐Tet21N cells overexpressing MYCN, in contrast to uninduced SHEP‐Tet21N cells where G1 arrest was pronounced (Figure 6C). Interestingly however, MYCN‐induced SHEP‐Tet21N cells exhibited a significant decrease of cells in G2/M‐phase after PRMT5 knockdown (*P* < 0.005), concomitant with an increase of cells in S‐phase. A similar effect on S‐phase has been demonstrated for c‐myc in medulloblastoma cells (Zhang et al., 2006). Together these experiments strongly support PRMT5 being able to directly influence MYCN protein stability and modulate its biological activities.

### 3.4 Physical and functional interaction of PRMT5 and MYCN

**Figure 7:**
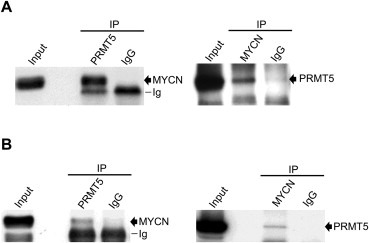
Physical interaction between endogenous PRMT5 and MYCN. (A) Co‐immunoprecipitation (IP) of MYCN with anti‐PRMT5 antibody (left) and the reciprocal co‐immunoprecipitation (right) in NGP cells. (B) Co‐immunoprecipitations as above in SK‐N‐BE(2)C cells.

We next sought to establish whether endogenous PRMT5 physically associated with MYCN complexes in NGP and SK‐N‐BE(2)C cells, as such an interaction may serve to stabilise MYCN and provide initial evidence that MYCN might be a substrate for the arginine methyltransferase activity of PRMT5. As shown in Figure 7A, MYCN was easily detected in PRMT5 complexes immunoprecipitated from NGP extracts and PRMT5 was also confirmed in the reciprocal MYCN immunoprecipitation. This robust interaction was also apparent in SK‐N‐BE(2)C cells (Figure 7B), and is consistent with the notion that PRMT5 is capable of stabilizing MYCN at the protein level.

**Figure 8:**
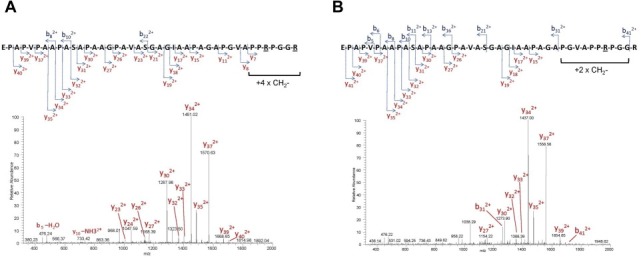
Detection of dimethylated arginine residues in MYCN. (A) LC‐MS/MS MS/MS spectrum of the doubly dimethylated peptide, EPAPVPAAPASAPAAGPAVASGAGIAAPAGAPGVAPPRPGGR (m/z 1211.66, 3+) from MYCN immunoprecipitated after control siRNA knockdown. The majority of ions are due to doubly charged C‐terminal y ions, which show that the two C‐terminal arginine residues are dimethylated (underlined arginines are dimethylated). (B) MS/MS spectrum of the singly dimethylated peptide EPAPVPAAPASAPAAGPAVASGAGIAAPAGAPGVAPPRPGGR (m/z 1202.30, 3+) from MYCN immunoprecipitated after PRMT5 knockdown. The doubly charged C‐terminal fragment ions in this spectrum are 14 m/z units (corresponding to a mass difference of 28 Da) lower than those shown in (A) as a result of the absence of a second dimethylated arginine residue, corresponding to R242 in MYCN (see Figure S5).

Lastly, we assessed whether MYCN protein may be methylated by PRMT5 using liquid chromatography – tandem mass spectrometry (LC‐MS/MS) analysis of immunoprecipitated MYCN protein from SK‐N‐BE(2)C cells before and after PRMT5 knockdown. This analysis gave us >53% coverage of MYCN, and identified several potential sites of arginine monomethylation and dimethylation on the MYCN protein (Figure S5). By inspecting spectra of MYCN peptides bioinformatically predicted to contain arginine methylation, we pinpointed R160, R238 and R242 as high probability sites for dimethylation. Discerning asymmetric and symmetric dimethylation was not possible from LC‐MS/MS analysis. However, by manually comparing the spectra for the peptide containing R238 and R242, from control siRNA and PRMT5 siRNA treated samples, we were able to identify fragment ions with reduced mass in the PRMT5 knockdown sample, consistent with the loss of dimethylarginine at R242 (Figure 8). The double‐dimethylated peptide shown in Figure 8A was not found in MYCN from PRMT5 knockdown, consistent with R242 representing a primary target for PRMT5 methyltransferase activity. However, it is important to note that our analysis was not quantitative and would therefore not allow us to identify subtler changes occurring between samples. Nevertheless, these experiments demonstrate for the first time that MYCN undergoes PTM by arginine methylation, and mechanistically implicate PRMT5 as a critical regulator of MYCN protein levels.

## 4 Conclusion

PRMT5 has pleiotropic regulatory roles in the cytoplasm and nucleus in normal cells, and has been shown to be crucial for normal development (Karkhanis et al., 2011), emphasising the importance of defining PRMT5 substrates in normal and cancer cells. Here we show for the first time that MYCN is a novel PRMT5 target that is regulated by post‐translational arginine methylation. The interplay of MYCN methylation and phosphorylation in regulating MYCN remains to be elucidated. As MYCN is an oncogenic driver in a wide variety of cancers, our findings highlight the importance of developing PRMT5 small molecule inhibitors for cancer therapeutics targeting MYCN; currently no specific inhibitors are available. Additionally, our data on *NGFR* reactivation by PRMT5 knockdown also suggest that PRMT5 is a co‐operative co‐repressor for MYCN‐mediated gene regulation. This is also supported by the report that the gene encoding MEP50/WDR77, which is a key component necessary for PRMT5 enzymatic activity, is positively regulated by MYCN (Valentijn et al., 2012). Furthermore, this raises the intriguing possibility that MYCN may have an additional regulatory mode involving directing global post‐translational modifications by arginine methylation. These possibilities are currently being examined using proteomic approaches in our laboratories.

## Conflict of interest

The authors declare no conflicts of interest.

## Supporting information



The following are the supplementary data related to this article:

Supplementary Table 1 Sequences of primers and short interfering RNAs used in this study, together with details of antibodies used for various applications.

Supplementary Figure S1 Absence of cell death and morphological changes in immortalized human fibroblasts, despite efficient PRMT5 knockdowns.

Supplementary Figure S2 (A) High PRMT5 mRNA expression correlates with MYCN amplification (MYCN‐amp) in NB. Data analysed is from Valentijn et al., 2012, using the R2 microarray analysis and visualization platform (http://r2.amc.nl). Tumours with no MYCN amplification (MYCN‐un) show lower PRMT5 mRNA expression. Kaplan Meier curve showing high PRMT5 mRNA expression correlates with poor prognosis. The Bonferroni corrected probability (Bonf P) is shown. (B) Kaplan Meier curve showing high PRMT5 mRNA expression correlates with poor prognosis in MYCN‐unamplified tumours.

Supplementary Figure S3 For PRMT5 immunohistochemistry controls, we used skeletal muscle which is negative for PRMT5 (left) and normal prostate where PRMT5 expression has been reported to be strong in the nucleus of the epithelial cells (Gu et al., 2012) (right). Weak staining in the blood vessels is indicated (ves), contrasting with the skeletal muscle (ske). Note the strong nuclear staining in prostate epithelial cells (epi) contrasting with the myoepithelium (myo).

Supplementary Figure S4 (A) Gene expression changes accompanying PRMT5 and MYCN knockdowns in NB cell‐lines. (B) Quantitative reverse transcriptase PCR analysis of MYCN expression after PRMT5 knockdown.

Supplementary Figure S5 MYCN protein sequence and peptide coverage by LC‐MS/MS is shown by shading and the number of peptides detected. Arginines predicted to be mono‐ and/or dimethylated are marked by red boxes, whereas the arginine predicted to be monomethylated is indicated by a stippled red box.

## Supplementary data 1

### 1.1

Supplementary data related to this article can be found at http://dx.doi.org/10.1016/j.molonc.2014.10.015.
