# Supplementary figures and images for: Protein arginine methyltransferase 5 is a key regulator of the MYCN oncoprotein in neuroblastoma cells
Source: Mol Oncol. 2014 Nov 15;9(3):617–27. doi: 10.1016/j.molonc.2014.10.015 (PMC4359099; doi:10.1016/j.molonc.2014.10.015)

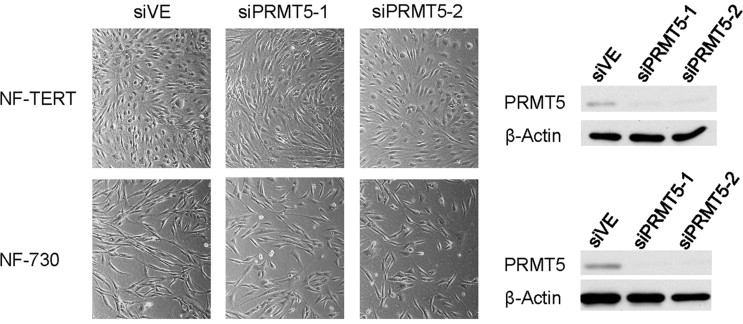

Supplement: Supplementary file 2 — Supplementary Figure S1 Absence of cell death and morphological changes in immortalized human fibroblasts, despite efficient PRMT5 knockdowns. [file MOL2-9-617-s001.jpg]

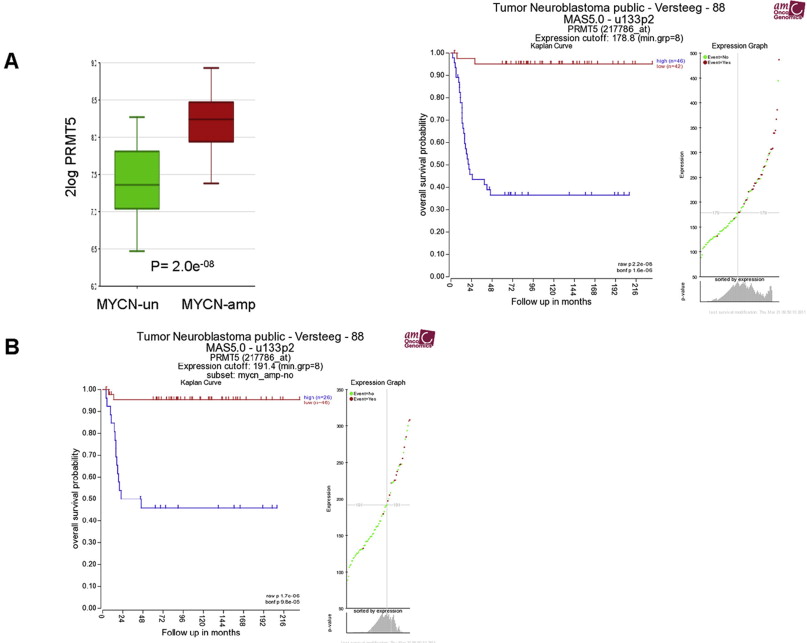

Supplement: Supplementary file 3 — Supplementary Figure S2 (A) High PRMT5 mRNA expression correlates with MYCN amplification (MYCN‐amp) in NB. Data analysed is from Valentijn et al., 2012, using the R2 microarray analysis and visualization platform (http://r2.amc.nl). Tumours with no MYCN amplification (MYCN‐un) show lower PRMT5 mRNA expression. Kaplan Meier curve showing high PRMT5 mRNA expression correlates with poor prognosis. The Bonferroni corrected probability (Bonf P) is shown. (B) Kaplan Meier curve showing high PRMT5 mRNA expression correlates with poor prognosis in MYCN‐unamplified tumours. [file MOL2-9-617-s002.jpg]

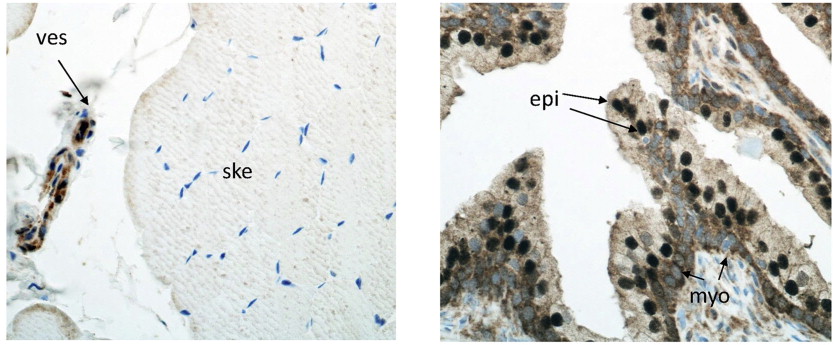

Supplement: Supplementary file 4 — Supplementary Figure S3 For PRMT5 immunohistochemistry controls, we used skeletal muscle which is negative for PRMT5 (left) and normal prostate where PRMT5 expression has been reported to be strong in the nucleus of the epithelial cells (Gu et al., 2012) (right). Weak staining in the blood vessels is indicated (ves), contrasting with the skeletal muscle (ske). Note the strong nuclear staining in prostate epithelial cells (epi) contrasting with the myoepithelium (myo). [file MOL2-9-617-s003.jpg]

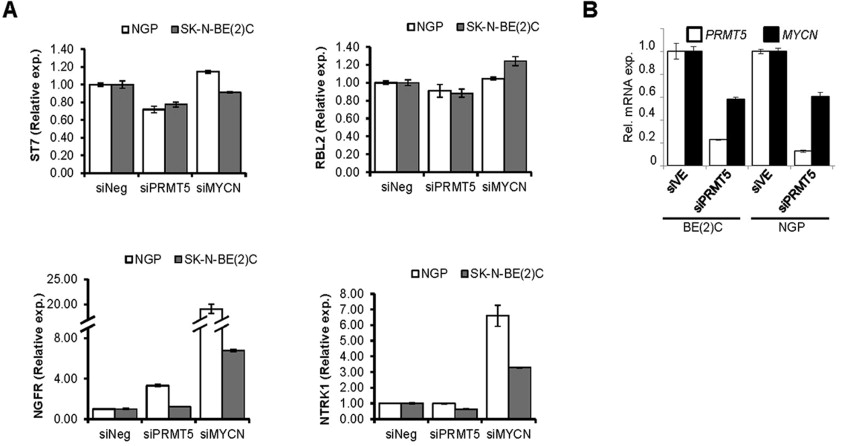

Supplement: Supplementary file 5 — Supplementary Figure S4 (A) Gene expression changes accompanying PRMT5 and MYCN knockdowns in NB cell‐lines. (B) Quantitative reverse transcriptase PCR analysis of MYCN expression after PRMT5 knockdown. [file MOL2-9-617-s004.jpg]

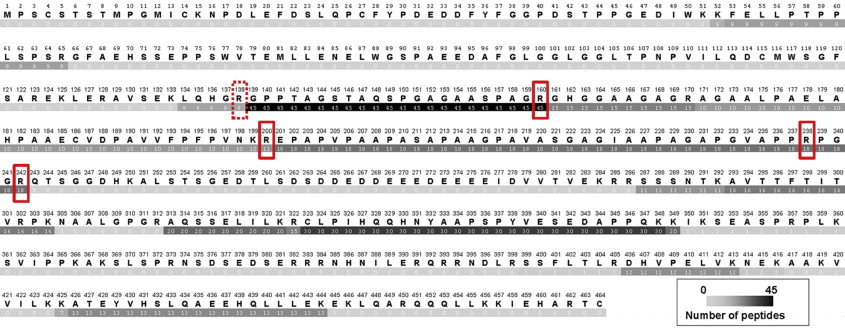

Supplement: Supplementary file 6 — Supplementary Figure S5 MYCN protein sequence and peptide coverage by LC‐MS/MS is shown by shading and the number of peptides detected. Arginines predicted to be mono‐ and/or dimethylated are marked by red boxes, whereas the arginine predicted to be monomethylated is indicated by a stippled red box. [file MOL2-9-617-s005.jpg]
